# Modulating the NLRP3/IL-1β Axis in Age-Related Atherosclerosis: Natural Products, Molecular Mechanisms, and Translational Perspectives—A Narrative Review

**DOI:** 10.3390/biom16101409

**Published:** 2026-09-28

**Authors:** Yelizaveta A. Marakhovskaya, Ksenia A. Eruslanova, Olga N. Maltseva, Zhengzhi Wu, Alexey V. Churov

**Affiliations:** Russian Gerontology Research and Clinical Centre, Pirogov Russian National Research Medical University (Pirogov University), Moscow 129226, Russia; eruslanova_ka@rgnkc.ru (K.A.E.); movolya@mail.ru (O.N.M.);

**Keywords:** atherosclerosis, inflammaging, interleukin-1β, NLRP3 inflammasome, natural products, traditional Chinese medicine

## Abstract

Atherosclerosis is an age-related immune-metabolic disease in which lipid retention, endothelial dysfunction, innate immune activation, and vascular remodeling interact over decades. In older adults, inflammaging, mitochondrial dysfunction, defective autophagy/mitophagy, and cellular senescence may lower the threshold for persistent inflammasome activation in the vascular wall. The NLR family pyrin domain containing 3 (NLRP3) inflammasome and interleukin-1β (IL-1β) are central to this process because they convert sterile danger signals, including cholesterol crystals, oxidized lipids, mitochondrial stress, and disturbed flow, into caspase-1 activation, IL-1β and interleukin-18 (IL-18) maturation, pyroptosis, endothelial dysfunction, macrophage activation, and plaque destabilization. Clinical proof-of-concept from IL-1β blockade supports inflammatory targeting in atherosclerotic cardiovascular disease, but direct cytokine inhibition leaves upstream inflammasome-prone states insufficiently addressed. This narrative review synthesizes evidence published from 2010 to 2026, together with older landmark studies, on mechanistic, translational, and clinical links between the NLRP3/IL-1β axis and age-related atherosclerosis. Traditional Chinese medicine-derived formulas and isolated natural compounds are considered because they may modulate upstream priming, mitochondrial reactive oxygen species, autophagy, lipid handling, macrophage polarization, endothelial pyroptosis, and inflammasome-associated cytokine release. However, most data remain preclinical, and translation requires stronger causal validation, chemical standardization, pharmacokinetic characterization, age-relevant models, and systematic safety assessment.

## 1. Introduction

Atherosclerosis is the principal pathological substrate of ischemic heart disease, ischemic stroke, peripheral artery disease, and other major manifestations of cardiovascular disease. Its clinical importance is increasing as population aging changes the structure of cardiovascular risk worldwide. Global analyses consistently identify cardiovascular disease as a leading contributor to mortality and disability, while age remains one of the strongest determinants of atherosclerotic burden and clinical events [1,2,3]. Elderly patients often accumulate traditional risk factors such as dyslipidemia, hypertension, diabetes, and chronic kidney disease, but aging also modifies the biology of the arterial wall itself. Recent reviews have further emphasized that disturbances in lipid, glucose, and amino acid metabolism contribute to atherosclerosis progression and may interact with inflammatory remodeling of the vascular wall [4]. Atherosclerosis is therefore best understood as a chronic inflammatory and immune-metabolic disease in which lipid retention, endothelial dysfunction, leukocyte recruitment, macrophage activation, vascular smooth muscle cell remodeling, and plaque destabilization interact over decades [5,6].

Population aging makes this biological framing particularly important. The number and proportion of elderly individuals are increasing globally, and this demographic shift is expected to expand the burden of chronic cardiovascular disease, multimorbidity, frailty, and polypharmacy [7,8]. Aging is associated with persistent low-grade inflammatory activation, impaired resolution of inflammation, mitochondrial dysfunction, altered autophagy and mitophagy, and accumulation of senescent cells with a senescence-associated secretory phenotype [9,10,11]. These changes lower the threshold for innate immune activation in vascular and immune cells and help explain why plaque progression and plaque vulnerability depend not only on lipid burden but also on the persistence of local inflammatory circuits.

Cytokines are central mediators of this inflammatory vascular environment. In parallel with cytokine signaling, neutrophil extracellular trap formation (NETosis), cathelicidin-related innate immune responses, and regulatory T-cell-dependent immune control represent additional mechanisms through which immune imbalance can promote or restrain atherosclerotic inflammation [12,13,14]. Cytokines promote endothelial activation, monocyte recruitment, macrophage polarization, foam-cell formation, vascular smooth muscle cell phenotypic switching, extracellular matrix remodeling, and thinning of the fibrous cap [5,6]. Within this network, the interleukin-1 (IL-1) family has a distinct position because it connects sterile danger sensing to downstream inflammatory amplification. IL-1α may contribute to early leukocyte adhesion and local vascular activation, whereas interleukin-1β (IL-1β) is especially relevant because it is produced predominantly by activated myeloid cells and can amplify endothelial dysfunction, macrophage inflammatory activation, pyroptotic cell death, and plaque destabilization [15,16,17,18].

The biological activity of IL-1β depends on regulated maturation through inflammasome-dependent processing. The NLR family pyrin domain containing 3 (NLRP3) inflammasome is one of the best-characterized platforms responsible for this conversion in sterile inflammatory diseases and atherosclerosis [15,19,20,21]. Its priming step is commonly mediated by Toll-like receptor or IL-1 receptor signaling and nuclear factor kappa B activation, whereas its activation step can be triggered by cholesterol crystals, mitochondrial reactive oxygen species, oxidized mitochondrial DNA, lysosomal rupture, ionic fluxes, and other metabolic danger signals. Assembly of the NLRP3 inflammasome then promotes caspase-1 activation, maturation of IL-1β and interleukin-18 (IL-18), gasdermin D cleavage, and pyroptosis [15,20,21,22].

This NLRP3/IL-1β pathway provides a bridge between inflammaging and atherosclerosis. Age-related mitochondrial injury, impaired autophagic clearance, senescence-associated inflammatory programs, and macrophage immunometabolic remodeling can make the aging vascular wall more permissive to inflammasome priming and IL-1β release [9,22,23,24]. Clinical evidence from canakinumab in patients with previous myocardial infarction and residual inflammatory risk further supports the pathogenic relevance of IL-1β in atherosclerotic cardiovascular disease, although direct cytokine blockade is limited by cost, parenteral administration, infection risk, and heterogeneous responses [18,22].

These limitations have created interest in strategies that modulate inflammatory signaling upstream or in parallel with IL-1β. Natural product-based interventions, including formulas and compounds derived from traditional Chinese medicine, may be relevant because they can affect several interrelated biological nodes rather than a single cytokine. Experimental studies suggest that selected formulas and compounds influence Toll-like receptor/nuclear factor kappa B-dependent priming, mitochondrial oxidative stress, autophagy and mitophagy, macrophage polarization, endothelial pyroptosis, lipid handling, and inflammasome-associated cytokine release in atherosclerosis-relevant models [25,26,27,28,29,30]. However, their translational value depends on evidence strength, chemical standardization, dose reproducibility, pharmacokinetics, safety, and validation in age-relevant models, particularly because elderly patients frequently receive antiplatelet agents, anticoagulants, statins, antihypertensive drugs, and glucose-lowering therapies [31,32,33].

This review aims to synthesize current evidence on natural product-based modulation of the NLRP3/IL-1β axis in age-related atherosclerosis, with particular attention to traditional Chinese medicine-derived formulas and compounds. The review focuses on the role of IL-1β and NLRP3 in linking inflammaging to plaque inflammation, evaluates the evidence for candidate interventions, and discusses the main translational barriers, including preclinical model limitations, standardization, and safety considerations relevant to elderly patients.

## 2. Search Strategy and Selection Criteria

This narrative review was developed through a focused literature search aimed at identifying mechanistic, translational, and clinical evidence linking the NLRP3/IL-1β axis, inflammaging, atherosclerosis, and natural product-based interventions. PubMed/MEDLINE was used as the primary biomedical database, with additional records identified through citation chaining, publisher pages, and targeted searches on traditional Chinese medicine, Chinese herbal formulas, and natural products relevant to atherosclerotic inflammation. The search covered publications from 2010 to 2026; older landmark studies were included when they established key mechanisms, such as cholesterol crystal-induced NLRP3 inflammasome activation or clinical validation of IL-1β blockade. Search terms included combinations of atherosclerosis, cardiovascular disease, vascular aging, inflammaging, cellular senescence, NLRP3, inflammasome, IL-1β, pyroptosis, macrophage, endothelium, traditional Chinese medicine, Chinese herbal medicine, herbal formula, natural product, phytochemical, and herb–drug interaction. Priority was given to peer-reviewed mechanistic studies, clinical trials, high-quality reviews, and experimental studies with direct relevance to IL-1/NLRP3 signaling, vascular aging, or natural product-based inflammasome modulation. Studies with only nonspecific anti-inflammatory findings, unclear bibliographic information, or insufficient disease/model relevance were not used as primary support; preclinical studies were interpreted as evidence of biological plausibility rather than proof of clinical efficacy.

## 3. IL-1/NLRP3 Signaling as an Effector Axis in Plaque Inflammation

The IL-1 family includes pro-inflammatory cytokines, receptor antagonists, decoy receptors, and inhibitory receptors that together regulate the initiation and resolution of inflammation. For the present review, the most relevant distinction is between IL-1α and IL-1β. IL-1α can act as an alarmin during cellular injury and may contribute to early leukocyte adhesion, whereas IL-1β is produced as an inactive precursor and becomes biologically active only after proteolytic processing, most commonly through inflammasome-associated caspase-1 activation [17,34,35]. This maturation requirement makes IL-1β a particularly useful point of connection between sterile danger sensing and atherosclerotic inflammation.

In atherosclerotic plaques, the NLRP3 inflammasome translates metabolic and structural injury into IL-1β-dependent inflammatory signaling. Cathelicidin biology is also relevant to cardiovascular inflammation because LL-37/cathelicidin-related antimicrobial peptide (CRAMP) can interact with lipoproteins and mitochondrial DNA and has been linked to Toll-like receptor 4- and NLRP3-related inflammatory signaling in cardiovascular contexts [13]. Cholesterol crystals, oxidized lipoproteins, lysosomal damage, mitochondrial reactive oxygen species, potassium efflux, and mitochondrial DNA release can promote NLRP3 activation in macrophages and vascular cells [15,19,20,21]. Once activated, the NLRP3 inflammasome complex, composed of NLRP3, apoptosis-associated speck-like protein containing a CARD (ASC), and caspase-1, promotes maturation of IL-1β and IL-18 and cleavage of gasdermin D, thereby coupling cytokine release to pyroptotic cell death. In this setting, inflammasome activation is not a generic inflammatory event; it is a mechanism by which retained lipids and cellular stress are converted into sustained immune communication within the plaque.

IL-1β amplifies several cellular processes that are directly relevant to plaque progression and vulnerability. In endothelial cells, IL-1 signaling increases adhesion molecule expression and chemokine production, thereby facilitating monocyte recruitment. In macrophages, it reinforces inflammatory activation, promotes further cytokine release, and can accompany pyroptotic cell death. In vascular smooth muscle cells, IL-1 signaling may favor phenotypic switching, inflammatory remodeling, and changes in extracellular matrix homeostasis, all of which can weaken the structural stability of the fibrous cap [6,22,36]. Thus, IL-1β should be interpreted not only as a marker of vascular inflammation but also as an active mediator that links lipid accumulation, innate immune activation, and plaque destabilization.

The importance of this pathway is supported by both experimental and clinical evidence. Genetic and pharmacological studies have shown that NLRP3 inflammasome activation contributes to atherogenesis and plaque inflammation, while the Canakinumab Anti-Inflammatory Thrombosis Outcomes Study demonstrated that selective IL-1β blockade can reduce recurrent cardiovascular events in patients with previous myocardial infarction and residual inflammatory risk [15,18,19]. At the same time, the clinical history of IL-1-targeted therapy also shows that biological plausibility does not automatically translate into broad therapeutic utility. Infection risk, treatment cost, parenteral administration, biomarker-based patient selection, and heterogeneous responses limit the use of direct cytokine blockade as a general strategy for atherosclerotic disease. The clinical evidence and its translational implications are summarized in Table 1.

This distinction is important for the logic of integrative and natural product-based interventions. If IL-1β production in plaques depends on upstream priming, mitochondrial stress, defective autophagic clearance, lipid-induced danger signals, and pyroptotic readiness, then interventions do not need to block IL-1β directly to influence this axis. They may instead reduce the availability of inflammasome-activating signals, dampen nuclear factor kappa B-dependent priming, improve mitochondrial stress responses, normalize autophagy or mitophagy, or shift macrophage and endothelial cells away from an inflammasome-prone state. This broader framing provides the rationale for evaluating traditional Chinese medicine-derived formulas and compounds as modulators of the NLRP3/IL-1β axis rather than as nonspecific anti-inflammatory agents.

## 4. Traditional Chinese Medicine as a Modulator of the NLRP3/IL-1β Axis in Atherosclerosis

Traditional Chinese Medicine (TCM)-derived bioactive compounds differ from single-target pharmacology. TCM formulas combine herbs with partially overlapping, yet non-identical, spectra of biological activity. Accordingly, the key question is not whether TCM-derived bioactive compounds “work” in a general sense but whether specific formulas or monomers can reproducibly modulate inflammasome biology and IL-1β signaling in atherosclerosis models. Experimental studies in which traditional Chinese medicine-derived formulas or isolated natural products modulate the NLRP3/caspase-1/IL-1β axis in atherosclerosis-relevant models are summarized in Table 2. This distinction is important because the issue at hand is not nonspecific anti-inflammatory activity, but modulation of innate immune signaling that converts metabolic injury of the vascular wall into a sustained inflammatory response.

Among the agents summarized in Table 2, Xinmaikang is one of the most thoroughly characterized. In this study, its effects were associated not only with a reduction in lesion area and improvement in the lipid profile, but also with sequential suppression of SREBP2, NLRP3, ASC, caspase-1, and IL-1β, both in vivo and in bone marrow-derived macrophages. Importantly, the authors did not restrict their analysis to descriptive associations; they employed small interfering RNA (siRNA)-mediated interference targeting SREBP2 and nodes of the inflammasome cascade, thereby demonstrating that the anti-atherosclerotic effect of Xinmaikang is mediated through SREBP2-dependent control of the inflammasome. In the context of atherosclerosis, this means that macrophages cease to function as cells that continuously support IL-1β maturation and instead shift toward a less inflammatory state, with immediate consequences for both the vascular wall and plaque morphology [25].

Resibufogenin acts directly on the inflammasome. In this case, direct inhibition of NLRP3 was demonstrated, and the study extended well beyond a mere reduction in IL-1β and IL-18 levels in the aortic tissue. The authors showed reduced ASC oligomerization, decreased cleaved caspase-1, diminished macrophage infiltration, and smaller atherosclerotic lesions, while also confirming the role of NLRP3 through in vitro overexpression/knockdown approaches and in vivo adeno-associated virus serotype 9 carrying short hairpin RNA against NLRP3 (AAV9-shNLRP3) experiments. This experimental design is particularly valuable for the present topic because it allows resibufogenin to be regarded as a compound that interferes with the assembly of the inflammasome, thereby reducing the availability of IL-1β within the plaque (Table 2) [42].

Corilagin occupies an important position in Table 2 because it links IL-1β-dependent inflammation to the macrophage sensory apparatus. In the experimental setting, corilagin reduced the expression of NLRP3, ASC, caspase-1, NIMA-related kinase 7 (NEK7), GSDMD, IL-1β, and IL-18, while simultaneously attenuating pyroptosis and inflammatory polarization of macrophages. This effect was tied to the olfactory receptor 2/adenylate cyclase 3 (Olfr2/Adcy3) axis, with causality tested using Olfr2 short hairpin RNA (shRNA) and Olfr2 overexpression. These findings support the interpretation that corilagin acts on an upstream sensory mechanism in vascular macrophages rather than merely reducing downstream cytokine markers [29].

Gentiopicroside acts predominantly at the level of the vascular endothelium. In apolipoprotein E-deficient (ApoE−/−) mice and human aortic endothelial cells (HAECs), it reduced IL-1β, IL-18, NLRP3, ASC, cleaved caspase-1, and cleaved GSDMD, while simultaneously attenuating ROS-dependent injury. Most importantly, this effect was linked to the sirtuin 1 (SIRT1)/nuclear factor erythroid 2-related factor 2 (Nrf2) pathway, and its relevance was confirmed by reversal following treatment with SIRT1 and Nrf2 inhibitors. This logic aligns particularly well with the biology of vascular aging: endothelial cells exposed to chronic oxidative stress become more permissive to inflammasome activation, whereas gentiopicroside effectively disrupts the connection between ROS and NLRP3 assembly. Consequently, both the inflammatory signal itself and the endothelial propensity to sustain chronic vascular activation are reduced (Table 2).

The combination of geniposide and notoginsenoside R1 (NGR1) represents a different type of intervention, in which IL-1β-dependent inflammation is reduced by modulating endothelial energy- and stress-response pathways. In that study, the expression of NLRP3, caspase-1, IL-1β, and IL-18 was reduced in both the aorta and human umbilical vein endothelial cells (HUVECs), along with improvement in plaque status and attenuation of endothelial apoptosis. This effect was linked to the activation of the AMP-activated protein kinase (AMPK)/mechanistic target of rapamycin (mTOR)/Nrf2 pathway, and the causal role of this signaling axis was confirmed by pharmacological rescue using 5-aminoimidazole-4-carboxamide ribonucleotide (AICAR) and dorsomorphin. In this model, IL-1β appears as part of a broader endothelial stress response, which is precisely why the geniposide–NGR1 combination is particularly informative; it shows how the modulation of cellular energy homeostasis can reduce IL-1β availability and thereby slow atherogenesis (Table 2).

NGR1 alone is of interest because it reproduces part of these effects as a single monomer. In ApoE−/− mice and oxidized low-density lipoprotein (oxLDL)-stimulated macrophages, it reduced the expression of components of the NLRP3/caspase-1/ASC complex, as well as IL-1β and IL-18, while attenuating foam cell formation and the severity of atherosclerotic lesions. Although the causality of the axis was not validated here as rigorously as in the Xinmaikang or resibufogenin studies, this study remains important because it places NGR1 at the center of the macrophage inflammatory cascade. In other words, this compound appears to influence not only lipid handling but also the very capacity of the macrophage to sustain IL-1β-dependent inflammation within the plaque (Table 2) [30].

Aloperine occupies an intermediate position between direct inflammasome inhibitors and compounds that primarily dampen the pyroptotic readiness of macrophages. In ApoE−/− mice and in cell-based systems, it reduced IL-1β and IL-18 levels, decreased cleaved caspase-1, and suppressed macrophage pyroptosis. Although the study indicates that the effect of aloperine is linked to inflammasome activation, it does not establish pathway causality to the same degree as studies employing explicit genetic perturbation. Nevertheless, it remains relevant because it shows that even without direct pharmacologic blockade of NLRP3, IL-1β-dependent macrophage-driven plaque injury can still be attenuated through suppression of pyroptosis (Table 2) [43].

Tongxinluo highlights another level of relevance for atherosclerosis, namely endothelial pyroptosis. In this study, the formula reduced ROS, dampened NLRP3/caspase-1 signaling, and mitigated endothelial injury, accompanied by slower atherosclerosis progression in ApoE−/− mice. The IL-1β axis is involved as part of the inflammasome–pyroptotic response of the endothelium, although in the currently accessible primary description, it was not validated as the single indispensable node. Even so, the biological significance of this observation is considerable: the endothelial cell does not merely respond to IL-1β but can itself sustain inflammasome activity, thereby amplifying leukocyte recruitment and injury to the vascular lining. Tongxinluo therefore acts not only on inflammation in general but specifically on that form of endothelial cell death and activation that supports plaque progression (Table 2).

The combination of NGR1 and saikosaponin B2 is presented in Table 2 as a more preliminary yet still meaningful entry. The main emphasis is placed on macrophage autophagy and its connection to NLRP3-dependent inflammatory signaling. The authors demonstrated a reduction in IL-1β and suppression of the NLRP3-related inflammatory cascade, linking this effect to modulation of phosphoinositide 3-kinase (PI3K)/AKT/mTOR signaling and restoration of autophagic control. Although the canonical ASC/caspase-1 axis was not fully validated in this study, it complements the overall logic well: in the aging vessel, impaired autophagy renders the macrophage more prone to inflammasome activation, and compounds capable of normalizing this layer of regulation may therefore reduce IL-1β availability as well.

Two formula-based studies further broaden this pattern. Guizhitongluo Tablet links macrophage pyroptosis to Piezo1/NLRP3 signaling, with evidence from ApoE−/− mice and BMDM foam-cell models. Buyang Huanwu Decoction shifts the focus from a single vascular cell type to the plaque environment as a whole, connecting vulnerable plaque stabilization with gut microbiota regulation, Toll-like receptor 4 (TLR4)/myeloid differentiation primary response 88 (MyD88)/nuclear factor kappa B (NF-κB)/NLRP3 signaling, and mitophagy. These studies are useful for illustrating the multitarget logic of formula-based interventions, but they should be interpreted as preclinical evidence rather than as direct evidence of clinical efficacy.

Table 2 indicates that TCM-derived bioactive compounds in the treatment of atherosclerosis cannot be reduced to an abstract “anti-inflammatory” effect. Rather, distinct compounds and formulas intervene in specific cellular scenarios through which IL-1β becomes a persistent component of plaque biology. In macrophages, this primarily involves suppression of NLRP3, caspase-1, and pyroptosis; in endothelial cells, it involves attenuation of ROS-dependent inflammasome responses and restoration of stress resilience; and at the level of the vascular wall as a whole, it involves weakening of the intercellular signals that convert lipid accumulation into chronic inflammation. Figure 1 summarizes the pathway-level targets reported across these studies. Importantly, the in vivo experiments summarized in Table 2 used young adult mice or did not report animal age; none evaluated an aged atherosclerosis model.

## 5. Translational Limitations and Geroscience Perspectives

Although the evidence summarized in this review supports a link between atherosclerotic inflammation and the NLRP3/caspase-1/IL-1β axis, the translational value of TCM-derived compounds and formulas remains limited at present. Most studies summarized in Table 2 were performed in young ApoE−/− or LDLR−/− animals, whereas the central premise of this review is that inflammaging changes baseline vascular and immune biology. In older organisms, innate immune responsiveness, mitochondrial resilience, autophagic clearance, and senescence-associated secretory phenotype (SASP) signaling are already altered before experimental injury occurs. Therefore, findings obtained in young animals cannot be assumed to predict efficacy in older patients. A geroscience-oriented approach should ask whether an intervention modifies the age-conditioned vascular environment that permits inflammasome activation, rather than only whether it reduces plaque size in a conventional atherosclerosis model [17].

Another important limitation is the chemical and pharmacological heterogeneity of TCM-derived preparations. Some studies include LC-MS/MS or UPLC-based characterization, but batch-to-batch reproducibility, quantitative marker-compound control, pharmacokinetics, bioavailability, and tissue distribution remain unevenly reported. This issue is especially important when the claimed mechanism involves a defined inflammatory node such as NLRP3/IL-1β. For isolated compounds, a relationship between molecular target and vascular outcome can sometimes be tested directly; for multicomponent formulas, the same claim is harder to interpret because several mechanisms may operate simultaneously. Standardization is therefore not a technical detail but a condition for reproducible translation: without it, reduced IL-1β or NLRP3 expression in an experimental model may not identify the active driver of the vascular effect.

The evidence base also varies in depth. The strongest studies are those in which lower IL-1β is connected to defined pathways such as SREBP2, NLRP3, Olfr2/Adcy3, AMPK/Nrf2, Piezo1/NLRP3, or TLR4/MyD88/NF-κB/NLRP3, and in which pathway causality is tested through knockdown, overexpression, pharmacological rescue, binding assays, or convergent in vivo and in vitro endpoints. In other studies, reduced IL-1β or NLRP3 expression may be one component of a broader anti-inflammatory, lipid-lowering, antioxidant, or autophagy-modulating response. For translation, this distinction matters: future studies should establish whether plaque reduction truly depends on inflammasome/IL-1β modulation or whether this axis is a secondary marker of more general metabolic improvement [17].

From a geroscience perspective, the most promising feature of these interventions is not simply that they reduce IL-1β, but that they may act on upstream conditions that make the aging vascular wall susceptible to inflammasome activation. These include mitochondrial ROS, TLR4/MyD88/NF-κB-dependent priming, impaired autophagy and mitophagy, macrophage foam-cell stress, and endothelial pyroptosis. This framing positions natural product-based and TCM-derived interventions as potential modulators of vascular stress resilience rather than as nonspecific anti-inflammatory agents. However, this interpretation remains a hypothesis for translation until it is tested in aged models and in clinically relevant populations with residual inflammatory risk [17,42].

At the same time, the clinical transition of TCM-derived bioactive compounds into the field of atherosclerosis will be determined not only by biological logic but also by safety under conditions of polypharmacy, which is of fundamental importance for the older population. Older patients with atherosclerosis rarely receive a single agent; much more often, they are already taking statins, antiplatelet agents, anticoagulants, antihypertensive drugs, and glucose-lowering therapies. In such a setting, even compounds with convincing experimental anti-IL-1β activity must be evaluated through the lens of potential herb–drug interactions. This applies particularly to agents derived from Danshen, which may theoretically increase hemorrhagic risk when combined with antiplatelet or anticoagulant therapy, as well as to compounds with potential effects on transporters and cytochrome P450 (CYP)-dependent metabolism. The problem here lies not only in the frequency of such interactions but also in the fact that older patients are especially vulnerable to their clinical consequences because of age-related pharmacokinetic changes and the accumulation of comorbidities. Contemporary reviews of herbal–cardiovascular drug interactions emphasize that, for such agents, safety cannot be treated as a secondary issue after efficacy; rather, in the setting of cardiovascular polypharmacy, the interaction profile becomes part of the translational evaluation of the intervention itself [31,32,33].

A more rigorous translational framework is therefore needed. Future studies should include aged animals of both sexes, chemically standardized formulas or well-characterized compounds, dose-exposure assessment, comparison with established anti-inflammatory strategies, and early clinical studies in patients selected by inflammatory risk. Endpoints should include not only lipid profile or plaque size but also inflammasome-related biomarkers, endothelial and macrophage phenotypes, plaque stability, safety, and interaction risk. Under these conditions, it may become possible to determine whether natural product-based and TCM-derived interventions merely accompany reduced IL-1β or can genuinely modify the age-associated inflammatory circuit through which aging accelerates atherosclerosis. The principal translational opportunities, current limitations, and required next steps are summarized in Table 3.

## 6. Conclusions

The present review indicates that atherosclerosis should be viewed not only as a disorder of lipid accumulation, but also as a consequence of sustained changes in innate immune regulation. In the aging vascular wall, mitochondrial dysfunction, impaired autophagic control, endothelial stress, macrophage activation, and SASP-related signaling create conditions in which inflammasome activation becomes easier to initiate and harder to resolve. Under these conditions, the NLRP3/caspase-1/IL-1β axis may act as one of the key molecular links between inflammaging and the persistence of vascular inflammation.

From this perspective, IL-1β is not simply an isolated pro-inflammatory cytokine. It participates in a broader inflammatory process that connects endothelial activation, leukocyte recruitment, macrophage pyroptosis, extracellular matrix remodeling, and plaque destabilization. This makes the NLRP3/IL-1β axis a biologically meaningful target for understanding how aging modifies the course of atherosclerosis and why residual inflammatory risk remains clinically important even when lipid levels are controlled.

The data summarized in this review suggest that selected TCM-derived formulas and isolated natural compounds may influence this inflammatory axis at several levels. Their effects involve suppression of NLRP3 inflammasome activation, reduction in IL-1β and IL-18 release, attenuation of oxidative stress, modulation of autophagy, and protection against endothelial or macrophage pyroptosis. However, these effects should not be interpreted as evidence of established clinical efficacy. In most cases, the available data remain preclinical, and reduced NLRP3 or IL-1β expression may reflect either direct inflammasome modulation or a secondary consequence of broader metabolic, antioxidant, or anti-inflammatory activity.

Therefore, the main translational question is not whether TCM-derived bioactive compounds can reduce inflammatory markers in experimental models, but whether they can reproducibly modify the age-associated inflammatory mechanisms that contribute to plaque progression and instability. Future studies should use aged animals of both sexes, chemically standardized preparations, dose-exposure assessment, rescue approaches, and safety evaluation under conditions of cardiovascular polypharmacy. Only after such validation will it be possible to determine whether natural product-based and TCM-derived interventions can become rational complementary strategies for targeting inflammatory mechanisms in age-related atherosclerotic cardiovascular disease.

## Figures and Tables

**Figure 1:**
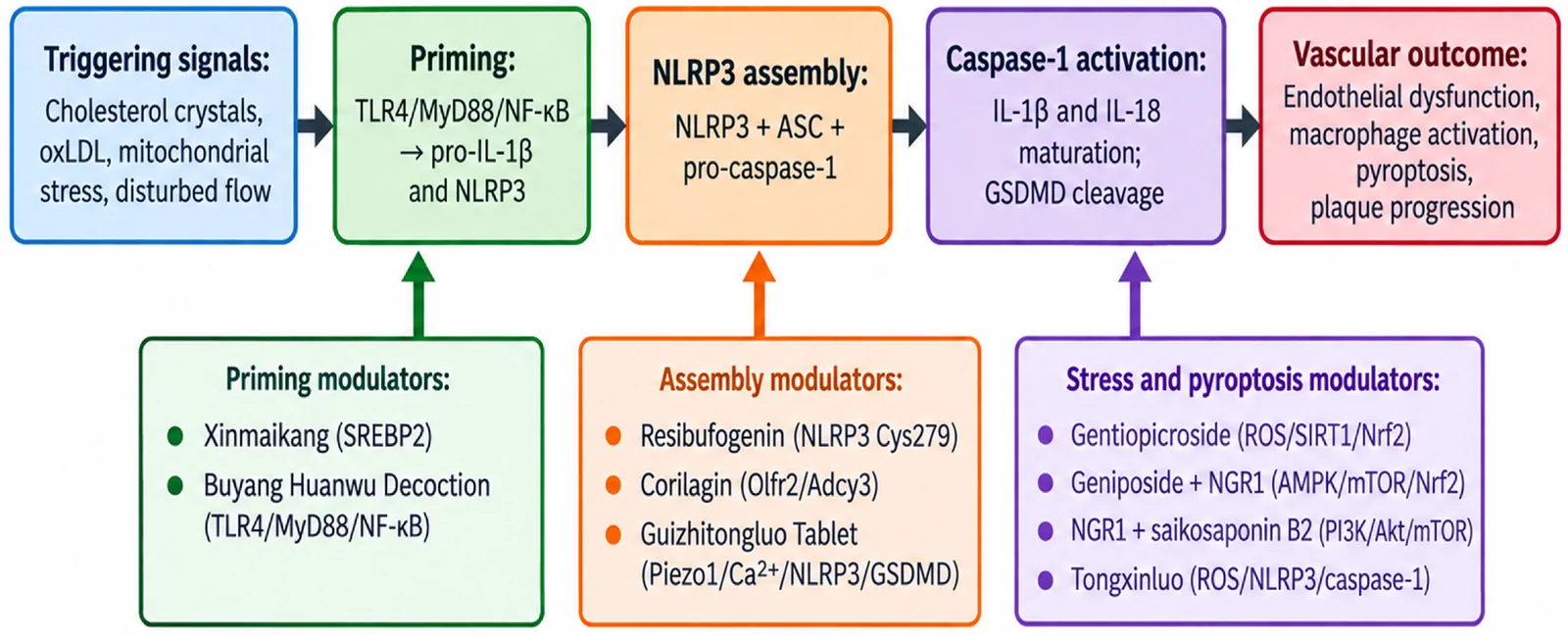
Natural products and TCM-derived formulas act at multiple nodes of the NLRP3/IL-1β axis. Pathway assignments summarize the experimental studies reported in Table 2 and do not establish clinical efficacy.

**Table 1:** Clinical proof-of-concept for inflammatory and IL-1 pathway targeting in atherosclerotic cardiovascular disease.

Intervention/Trial	Target/ Pathway	Population	Main Cardiovascular Findings	Limitation
Canakinumab/CANTOS	IL-1β	Prior MI, hsCRP ≥ 2 mg/L	Reduced recurrent CV events without lowering LDL-C	Infection risk, cost, parenteral therapy
Anakinra/VCU-ART series	IL-1 receptor	Acute MI/post-MI inflammation	Reduced inflammatory biomarkers; clinical event evidence limited	Small studies, surrogate endpoints
Colchicine/COLCOT or LoDoCo2	Inflammasome-related inflammation, not direct IL-1β blockade	Recent MI or chronic coronary disease	Reduced ischemic events in selected populations	Not specific to IL-1β/NLRP3; safety/tolerability issues

Abbreviations: CANTOS, Canakinumab Anti-Inflammatory Thrombosis Outcomes Study; COLCOT, Colchicine Cardiovascular Outcomes Trial; hsCRP, high-sensitivity C-reactive protein; IL-1β, interleukin-1 beta; LDL-C, low-density lipoprotein cholesterol; LoDoCo2, Low-Dose Colchicine 2; MI, myocardial infarction; VCU-ART, Virginia Commonwealth University Anakinra Remodeling Trial. Sources: [18,37,38,39,40,41].

**Table 2:** Natural product-based and traditional Chinese medicine-derived modulation of the NLRP3/IL-1β axis in experimental atherosclerosis.

Translational Limitation	Evidence Strength	Mechanism	NLRP3/IL-1β Effect	Target Cell	Model, Age, and Treatment Regimen	Compound/Formula
Multi-component formula; no human ASCVD outcome data	High preclinical: in vivo + BMDMs + siRNA/pathway testing	SREBP2-mediated regulation; SREBP2 knockdown reduced the XMK effect	Downregulated NLRP3, ASC, IL-1β, and caspase-1 mRNA/protein	Macrophages/BMDMs	ApoE−/− mice (age NR) + HFD 12 wk; XMK 2.28, 4.55, or 9.10 g/kg/day for 12 wk; LPS-stimulated BMDMs (1–40 μg/mL)	Xinmaikang tablets (XMK; Codonopsis pilosula [Dangshen], Citrus aurantium [Zhishi], Trionyx carapace [Biejia], and Pheretima [Dilong])
No human data; safety and exposure require study	High preclinical: in vivo + direct target-engagement evidence	Non-covalent interaction with NLRP3 Cys279; docking, SPR, pull-down, and knockdown studies	Inhibited NLRP3 inflammasome assembly and cytokine release	Macrophages/foam cells	ApoE−/− mice (age NR) + HFD 12 wk; oral 1, 3, or 5 mg/kg/day during weeks 5–12 (8 wk); cell, docking, and binding assays	Resibufogenin
No pharmacokinetic or clinical ASCVD evidence	High preclinical: in vivo + macrophage models + target gain-/loss-of-function	Olfr2/Adcy3 signaling; in vivo and in vitro Olfr2 gain-/loss-of-function experiments	Reduced macrophage NLRP3 activation and IL-1β release	Vascular macrophages	Male ApoE−/− mice, 5 wk at purchase (1-wk acclimatization); HF/HC diet 8 wk; 10, 20, or 40 mg/kg i.g. every 2 d for 2 wk; Ana-1 macrophages	Corilagin
No aged-animal or clinical evidence	High preclinical: in vivo + endothelial model + inhibitor-based support	SIRT1/Nrf2-dependent suppression of ROS and NLRP3 inflammasome activation	Reduced NLRP3 activation, caspase-1 activation, IL-1β release, and pyroptosis	Vascular endothelial cells	Male ApoE−/− mice, 8 wk; HFD 16 wk; gentiopicroside 40 mg/kg/day by gavage; oxLDL-treated HAECs	Gentiopicroside
Combination attribution and clinical exposure are unresolved	Moderate-high preclinical: in vivo + endothelial studies + pathway support	AMPK/mTOR/Nrf2 signaling	Reduced NLRP3, caspase-1, IL-1β, and endothelial pyroptosis	Vascular endothelial cells	Male ApoE−/− mice, 8 wk; HFD 12 wk, then geniposide 50 mg/kg/day, NGR1 50 mg/kg/day, or both for 12 wk i.p.; H_2_O_2_-treated HUVECs	Geniposide + notoginsenoside R1
Regimen reporting and clinical translation remain incomplete	Moderate preclinical: in vivo/cell evidence; causal pathway linkage remains limited	Network pharmacology plus experimental macrophage validation	Reduced NLRP3, caspase-1, and IL-1β	Macrophages	ApoE−/− mice + HFD; age, dose, and duration NR in the article abstract; oxLDL-induced macrophages	Notoginsenoside R1 (NGR1)
No aged-animal or clinical evidence	Moderate-high preclinical: in vivo + macrophage studies	Suppressed macrophage pyroptosis and inflammatory signaling	Reduced NLRP3 activation, caspase-1 activation, IL-1β release, and pyroptosis	Macrophages	ApoE−/− mice + Western diet (age NR); 40 mg/kg i.g. for 4 wk; THP-1-derived macrophages	Aloperine
Complex formula; active drivers and clinical exposure remain uncertain	Moderate-high preclinical: in vivo + endothelial studies	ROS/NLRP3/caspase-1-related signaling	Reduced NLRP3, caspase-1, IL-1β, and endothelial pyroptosis	Vascular endothelial cells	Male ApoE−/− mice, 7 wk at purchase (1-wk acclimatization); HFD 3 mo, then TXL 0.38, 0.75, or 1.50 g/kg/day by gavage for 3 mo; oxLDL-treated MAECs	Tongxinluo (TXL; 12-component formula including Panax ginseng, Hirudo, Scorpio, Paeoniae Radix Rubra, Scolopendra, and Borneolum Syntheticum)
Combination attribution and human dosing remain unresolved	Moderate-high preclinical: in vivo + macrophage studies + combination testing	PI3K/Akt/mTOR-associated autophagy regulation	Reduced NLRP3 activation, IL-1β release, and pyroptosis	Macrophages	Male ApoE−/− mice, 7 wk at purchase (1-wk acclimatization); Western diet 12 wk; NGR1 30 and/or saikosaponin B2 30 mg/kg/day i.p. during weeks 9–12; BMDMs	NGR1 + saikosaponin B2
Complex formula; no aged-animal or human outcome data	Moderate-high preclinical: in vivo + macrophage/pathway evidence	Piezo1/Ca^2+^/NLRP3/GSDMD signaling	Reduced NLRP3, ASC, cleaved caspase-1, IL-1β, GSDMD, and pyroptosis	Macrophages/BMDMs	ApoE−/− mice + HFD (age NR); 0.52, 1.04, or 2.08 g/kg/day by gavage for 8 wk; oxLDL/LPS-stimulated BMDMs treated with drug-containing serum	Guizhitongluo Tablet
Complex formula; reproducibility, pharmacokinetics, and clinical evidence are limited	Moderate preclinical: in vivo multitarget evidence; causal pathway linkage remains limited	Gut microbiota/metabolites, TLR4/MyD88/NF-κB/NLRP3 signaling, and mitophagy	Reduced NLRP3-associated inflammatory proteins and cytokines	Plaque environment and gut–vascular axis	ApoE−/− mice + HFD with choline chloride; age, dose, and duration NR in the article abstract	Buyang Huanwu Decoction

Evidence-strength criteria: ratings are qualitative, review-specific appraisals of preclinical mechanistic support and are not a clinical evidence grading system. High preclinical indicates an atherosclerosis model plus a mechanistic intervention, target-engagement experiment, or gain-/loss-of-function validation. Moderate-high preclinical indicates in vivo and relevant cell evidence with pharmacological or pathway-level support. Moderate preclinical indicates in vivo evidence with an indirect causal link to NLRP3/IL-1β. Age refers to age at study entry when reported; NR, not reported. Abbreviations: NR, not reported; AAV9, adeno-associated virus serotype 9; Adcy3, adenylate cyclase 3; AICAR, 5-aminoimidazole-4-carboxamide ribonucleotide; Akt, protein kinase B; ALO, aloperine; AMPK, AMP-activated protein kinase; ApoE−/−, apolipoprotein E-deficient; ASC, apoptosis-associated speck-like protein containing a CARD; ASCVD, atherosclerotic cardiovascular disease; BMDM/BMDMs, bone marrow-derived macrophage(s); Ca^2+^, calcium ion; EC, endothelial cell; GSDMD, gasdermin D; HAEC/HAECs, human aortic endothelial cell(s); HFD, high-fat diet; HO-1, heme oxygenase-1; HUVEC/HUVECs, human umbilical vein endothelial cell(s); IL-1β, interleukin-1 beta; IL-18, interleukin-18; JNK, c-Jun N-terminal kinase; LDLR−/−, low-density lipoprotein receptor-deficient; LPS, lipopolysaccharide; MAEC/MAECs, mouse aortic endothelial cell(s); mTOR, mechanistic target of rapamycin; MyD88, myeloid differentiation primary response 88; NEK7, NIMA-related kinase 7; NF-κB, nuclear factor kappa B; NGR1, notoginsenoside R1; NLRP3, NLR family pyrin domain containing 3; Nrf2, nuclear factor erythroid 2-related factor 2; Olfr2, olfactory receptor 2; oxLDL, oxidized low-density lipoprotein; PI3K, phosphoinositide 3-kinase; ROS, reactive oxygen species; shRNA, short hairpin RNA; SIRT1, sirtuin 1; SPR, surface plasmon resonance; SREBP2, sterol regulatory element-binding protein 2; TCM, traditional Chinese medicine; THP-1, human monocytic leukemia cell line; TLR4, Toll-like receptor 4; XMK, Xinmaikang. Sources: [25,26,27,28,29,30,42,43,44,45,46].

**Table 3:** Translational opportunities and current barriers for natural product-based and TCM-derived interventions in age-related atherosclerosis.

Domain	Translational Opportunity	Current Limitation/Required Next Step
Age-relevant biology	These interventions may act on upstream conditions that make the aging vascular wall susceptible to inflammasome activation, including mitochondrial ROS, impaired autophagy, endothelial pyroptosis, and macrophage stress.	Most evidence comes from young ApoE−/− or LDLR−/− animals. Validation in aged animals of both sexes is needed.
Specificity	The strongest studies connect IL-1β/NLRP3 reduction to defined pathways such as SREBP2, Olfr2/Adcy3, AMPK/Nrf2, Piezo1/NLRP3, or TLR4/MyD88/NF-κB/NLRP3.	Many studies still show marker reduction rather than proof that inflammasome modulation is required for plaque benefit.
Formula standardization	LC-MS/MS or UPLC-based profiling in some studies supports movement toward chemical characterization.	Batch reproducibility, marker-compound quantification, pharmacokinetics, bioavailability, and tissue distribution remain unevenly reported.
Multitarget action	Multitarget activity may be useful in age-related atherosclerosis, where lipid stress, oxidative injury, inflammation, endothelial dysfunction, and autophagy defects interact.	Multitarget effects complicate causal interpretation and make it difficult to identify the active driver of vascular benefit.
Clinical positioning	These interventions may complement, rather than replace, direct IL-1β blockade by acting upstream of inflammasome activation.	No clinical ASCVD outcome evidence is available for most candidates; early studies should focus on patients with residual inflammatory risk.
Safety in older adults	Translation should integrate efficacy with cardiovascular polypharmacy, comorbidity, and age-related pharmacokinetic variability.	Herb–drug interactions, especially with antiplatelet agents, anticoagulants, statins, and CYP/transport pathways, require systematic assessment.
Biomarker strategy	Inflammasome-related biomarkers could help connect preclinical mechanisms to clinical inflammatory risk.	Future studies should include hsCRP, IL-6, IL-1β/IL-18-related readouts, endothelial/macrophage phenotypes, and plaque-stability endpoints.

Abbreviations: Adcy3, adenylate cyclase 3; AMPK, AMP-activated protein kinase; ApoE−/−, apolipoprotein E-deficient; ASCVD, atherosclerotic cardiovascular disease; CYP, cytochrome P450; hsCRP, high-sensitivity C-reactive protein; IL-1β, interleukin-1 beta; IL-6, interleukin-6; IL-18, interleukin-18; LDLR−/−, low-density lipoprotein receptor-deficient; LC-MS/MS, liquid chromatography–tandem mass spectrometry; MyD88, myeloid differentiation primary response 88; NF-κB, nuclear factor kappa B; NLRP3, NLR family pyrin domain containing 3; Nrf2, nuclear factor erythroid 2-related factor 2; ROS, reactive oxygen species; SREBP2, sterol regulatory element-binding protein 2; TCM, traditional Chinese medicine; TLR4, Toll-like receptor 4; UPLC, ultra-performance liquid chromatography. Sources: [22,25,26,27,29,30,31,32,33,43,47].

## Data Availability

No new data were created or analyzed in this study. Data sharing is not applicable to this article.

## References

[1] Stark B.A., DeCleene N.K., Desai E.C., Hsu J.M., Johnson C.O., Lara-Castor L., LeGrand K.E., A B., Aalipour M.A., Aalruz H. (2025). Global, Regional, and National Burden of Cardiovascular Diseases and Risk Factors in 204 Countries and Territories, 1990–2023. J. Am. Coll. Cardiol..

[2] Tan S.C.W., Zheng B.B., Tang M.L., Chu H., Zhao Y.-T., Weng C. (2025). Global Burden of Cardiovascular Diseases and Its Risk Factors, 1990–2021: A Systematic Analysis for the Global Burden of Disease Study 2021. QJM.

[3] World Health Organization Cardiovascular Diseases Fact Sheet. https://www.who.int/news-room/fact-sheets/detail/cardiovascular-diseases-(cvds).

[4] Blagov A.V., Churov A.V., Golovyuk A.L., Lee A.A., Kashtalap V.V., Sukhorukov V.N., Orekhov A.N. (2024). The role of metabolic disorders in the development of atherosclerosis. Cell. Mol. Biol..

[5] Libby P., Buring J.E., Badimon L., Hansson G.K., Deanfield J., Bittencourt M.S., Tokgözoğlu L., Lewis E.F. (2019). Atherosclerosis. Nat. Rev. Dis. Primers.

[6] Wolf D., Ley K. (2019). Immunity and inflammation in atherosclerosis. Circ. Res..

[7] World Health Organization Ageing and Health Fact Sheet. https://www.who.int/news-room/fact-sheets/detail/ageing-and-health.

[8] North B.J., Sinclair D.A. (2012). The intersection between aging and cardiovascular disease. Circ. Res..

[9] Franceschi C., Garagnani P., Parini P., Giuliani C., Santoro A. (2018). Inflammaging: A new immune-metabolic viewpoint for age-related diseases. Nat. Rev. Endocrinol..

[10] Liberale L., Montecucco F., Tardif J.C., Libby P., Camici G.G. (2018). Inflammageing: Chronic inflammation in ageing, cardiovascular disease, and frailty. Nat. Rev. Cardiol..

[11] Ungvari Z., Tarantini S., Donato A.J., Galvan V., Csiszar A. (2018). Mechanisms of vascular aging. Circ. Res..

[12] Blagov A.V., Churov A.V., Starodubtseva I.A., Beloyartsev D.F., Kovyanova T.I., Pecherina T., Sukhorukov V.N., Orekhov A.N. (2024). The role of NETosis in enhancing of atherosclerosis. Cell Mol. Biol..

[13] Dabravolski S.A., Orekhov N.A., Churov A.V., Starodubtseva I.A., Beloyartsev D.F., Kovyanova T.I., Sukhorukov V.N., Orekhov A.N. (2024). Role of cathelicidins in atherosclerosis and associated cardiovascular diseases. J. Mol. Pathol..

[14] Churov A.V., Chegodaev Y.S., Khotina V.A., Ofitserov V.P., Orekhov A.N. (2023). Regulatory T cells in atherosclerosis: Is adoptive cell therapy possible?. Life.

[15] Rajamäki K., Lappalainen J., Öörni K., Välimäki E., Matikainen S., Kovanen P.T., Eklund K.K. (2010). Cholesterol Crystals Activate the NLRP3 Inflammasome in Human Macrophages: A Novel Link between Cholesterol Metabolism and Inflammation. PLoS ONE.

[16] Kirii H., Niwa T., Yamada Y., Wada H., Saito K., Iwakura Y., Asano M., Moriwaki H., Seishima M. (2003). Lack of interleukin-1beta decreases the severity of atherosclerosis in ApoE-deficient mice. Arterioscler. Thromb. Vasc. Biol..

[17] Maeder C., Speer T., Wirth A., Boeckel J.N., Fatima S., Shahzad K., Freichel M., Laufs U., Gaul S. (2023). Membrane-bound interleukin-1α mediates leukocyte adhesion during atherogenesis. Front. Immunol..

[18] Ridker P.M., Everett B.M., Thuren T., MacFadyen J.G., Chang W.H., Ballantyne C., Fonseca F., Nicolau J., Koenig W., Anker S.D. (2017). Antiinflammatory therapy with canakinumab for atherosclerotic disease. N. Engl. J. Med..

[19] Grebe A., Hoss F., Latz E. (2018). NLRP3 inflammasome and the IL-1 pathway in atherosclerosis. Circ. Res..

[20] Paik S., Kim J.K., Silwal P., Sasakawa C., Jo E.K. (2021). An update on the regulatory mechanisms of NLRP3 inflammasome activation. Cell Mol. Immunol..

[21] Xu J., Nunez G. (2023). The NLRP3 inflammasome: Activation and regulation. Trends Biochem. Sci..

[22] Toldo S., Abbate A. (2024). The role of the NLRP3 inflammasome and pyroptosis in cardiovascular diseases. Nat. Rev. Cardiol..

[23] Grootaert M.O.J., Moulis M., Roth L., Martinet W., Vindis C., Bennett M.R., De Meyer G.R.Y. (2018). Vascular smooth muscle cell death, autophagy and senescence in atherosclerosis. Cardiovasc. Res..

[24] Sharma B.R., Kanneganti T.D. (2021). NLRP3 inflammasome in cancer and metabolic diseases. Nat. Immunol..

[25] Hou C., Jiang X., Sheng W., Zhang Y., Lin Q., Hong S., Zhao J., Wang T., Ye X. (2024). Xinmaikang (XMK) tablets alleviate atherosclerosis by regulating the SREBP2-mediated NLRP3/ASC/caspase-1 signaling pathway. J. Ethnopharmacol..

[26] Jiang X., Ma C., Gao Y., Cui H., Zheng Y., Li J., Zong W., Zhang Q. (2023). Tongxinluo attenuates atherosclerosis by inhibiting ROS/NLRP3/caspase-1-mediated endothelial cell pyroptosis. J. Ethnopharmacol..

[27] Li Z.-q., Zhang F., Li Q., Wang L., Sun X.-q., Li C., Yin X.-m., Liu C.-l., Wang Y.-x., Du X.-y. (2025). Gentiopicroside alleviates atherosclerosis by suppressing reactive oxygen species-dependent NLRP3 inflammasome activation in vascular endothelial cells via SIRT1/Nrf2 pathway. Chin. J. Integr. Med..

[28] Liu X., Xu Y., Cheng S., Zhou X., Zhou F., He P., Hu F., Zhang L., Chen Y., Jia Y. (2021). Geniposide combined with notoginsenoside R1 attenuates inflammation and apoptosis in atherosclerosis via the AMPK/mTOR/Nrf2 signaling pathway. Front. Pharmacol..

[29] Mao J., Chen Y., Zong Q., Liu C., Xie J., Wang Y., Fisher D., Hien N.T.T., Pronyuk K., Musabaev E. (2024). Corilagin alleviates atherosclerosis by inhibiting NLRP3 inflammasome activation via the Olfr2 signaling pathway in vitro and in vivo. Front. Immunol..

[30] Yu J., Hu J., Baldini M., Lei H., Li L., Luo S., Wu J., Liu X., Shan D., Xie Y. (2024). Integrating network pharmacology and experimental models to identify notoginsenoside R1 ameliorates atherosclerosis by inhibiting macrophage NLRP3 inflammasome activation. J. Nat. Med..

[31] Chan E., Tan M., Xin J., Sudarsanam S., Johnson D.E. (2010). Interactions between traditional Chinese medicines and Western therapeutics. Curr. Opin. Drug Discov. Dev..

[32] Ge B., Zhang Z., Zuo Z. (2014). Updates on the clinical evidenced herb-warfarin interactions. Evid. Based Complement. Altern. Med..

[33] Teschke R., Eickhoff A. (2015). Herbal hepatotoxicity in traditional and modern medicine: Actual key issues and new encouraging steps. Front. Pharmacol..

[34] Gaballa J.M., Højen J.F., De Graaf D.M., Amo-Aparicio J., Marchetti C., Cavalli G., Dinarello A., Li S., Corbisiero M.F., Tengesdal I.W. (2024). International nomenclature guidelines for the IL-1 family of cytokines and receptors. Nat. Immunol..

[35] Garlanda C., Di Ceglie I., Jaillon S. (2025). IL-1 family cytokines in inflammation and immunity. Cell Mol. Immunol..

[36] Chen S., Savas A., Atici A., Lee Y., Lane M., Aubuchon E., Moreira D., Hsu-Wilbraham A., Shimada K., Crother T.R. (2025). Interleukin-1 signaling on vascular smooth muscle cells accelerates atherosclerosis in a murine model of Kawasaki disease. J. Am. Heart Assoc..

[37] Abbate A., Kontos M.C., Grizzard J.D., Biondi-Zoccai G.G.L., Van Tassell B.W., Robati R., Roach L.M., Arena R.A., Roberts C.S., Varma A. (2010). Interleukin-1 Blockade with Anakinra to Prevent Adverse Cardiac Remodeling after Acute Myocardial Infarction (Virginia Commonwealth University Anakinra Remodeling Trial [VCU-ART] Pilot Study). Am. J. Cardiol..

[38] Abbate A., Trankle C.R., Buckley L.F., Lipinski M.J., Appleton D., Kadariya D., Canada J.M., Carbone S., Roberts C.S., Abouzaki N. (2020). Interleukin-1 Blockade Inhibits the Acute Inflammatory Response in Patients with ST-Segment-Elevation Myocardial Infarction. J. Am. Heart Assoc..

[39] Nidorf S.M., Fiolet A.T.L., Mosterd A., Eikelboom J.W., Schut A., Opstal T.S.J., The S.H.K., Xu X.F., Ireland M.A., Lenderink T. (2020). Colchicine in patients with chronic coronary disease. N. Engl. J. Med..

[40] Ridker P.M., MacFadyen J.G., Everett B.M., Libby P., Thuren T., Glynn R.J., CANTOS Trial Group (2018). Relationship of C-Reactive Protein Reduction to Cardiovascular Event Reduction Following Treatment with Canakinumab: A Secondary Analysis from the CANTOS Randomised Controlled Trial. Lancet.

[41] Tardif J.C., Kouz S., Waters D.D., Bertrand O.F., Diaz R., Maggioni A.P., Pinto F.J., Ibrahim R., Gamra H., Kiwan G.S. (2019). Efficacy and safety of low-dose colchicine after myocardial infarction. N. Engl. J. Med..

[42] Chen X., Chen Y., Zhai C., Du W., Chen Z., Wang J., Xu X., Wang W., Li C. (2026). Resibufogenin protects against atherosclerosis in ApoE-/- mice through blocking NLRP3 inflammasome assembly. J. Adv. Res..

[43] Wang Z., Wang Y., Raza F., Zafar H., Guo C., Sui W., Yang Y., Li R., Fang Y., Li B. (2025). Aloperine alleviates atherosclerosis by inhibiting NLRP3 inflammasome activation in macrophages and ApoE-/- mice. Curr. Mol. Pharmacol..

[44] Wang Y., Liao Q., Mei X., Xu H., Luo L., Huang R., Tang Y., Zhou C. (2025). Synergistic effects of notoginsenoside R1 and saikosaponin B2 in atherosclerosis: A novel approach targeting PI3K/Akt/mTOR pathway and macrophage autophagy. PLoS ONE.

[45] Pan X., Xu H., Ding Z., Luo S., Li Z., Wan R., Jiang J., Chen X., Liu S., Chen Z. (2024). Guizhitongluo Tablet inhibits atherosclerosis and foam cell formation through regulating Piezo1/NLRP3-mediated macrophage pyroptosis. Phytomedicine.

[46] Chang S.Y., Li Y.T., Zhu H.Y., He Z.X., You Y., Liu Y.H. (2025). Buyang Huanwu Decoction stabilizes atherosclerotic vulnerable plaques by regulating intestinal flora, TLR4-NF-κB-NLRP3 inflammatory pathway and mitophagy. Phytomedicine.

[47] Lindkvist M., Göthlin Eremo A., Paramel G.V., Anisul Haque S., Rydberg Millrud C., Rattik S., Grönberg C., Liberg D., Sirsjö A., Fransén K. (2025). IL1RAP expression in human atherosclerosis: A target of novel antibodies to reduce vascular inflammation and adhesion. J. Am. Heart Assoc..

